# Preliminary Investigation into the Use of Amino-Acid-Derived Ionic Liquids for Extracting Cellulose from Waste Biomass to Prepare Cellulose Aerogel Adsorbents

**DOI:** 10.3390/gels11030210

**Published:** 2025-03-16

**Authors:** Yun Deng, Qiusheng Zhao, Shuai Nian, Ziyan Sha, Lin Fu, Ian Beadham, Xiaolan Xiao, Changbo Zhang

**Affiliations:** 1 College of Environment and Ecology, Jiangnan University, Wuxi 214122, China; dengyun@jiangnan.edu.cn (Y.D.); 6231402104@stu.jiangnan.edu.cn (Q.Z.); 18656202670@163.com (S.N.); 6231402018@stu.jiangnan.edu.cn (Z.S.); 8202101448@jiangnan.edu.cn (X.X.); 2 Key Laboratory of Original Agro-Environmental Pollution Prevention and Control, Ministry of Agriculture and Rural Affairs, Agro-Environmental Protection Institute, MARA, Tianjin 300191, China; fl1004197187@163.com; 3 School of Pharmacy and Chemistry, Kingston University, Kingston Upon Thames KT1 2EE, UK; i.beadham@kingston.ac.uk; 4 Institute of Future Food Technology, Jiangsu Industrial Technology Research Institute, Yixing 214200, China

**Keywords:** amino acid-based ionic liquids, cellulose aerogels, adsorption

## Abstract

To investigate the feasibility of cellulose extraction from lignocellulosic waste biomass using ionic liquids—a sustainable and efficient approach—for preparing cellulose aerogel adsorbents, we employed a fully green amino acid-derived ionic liquid, cysteine nitrate ([Cys][NO_3_]), for cellulose separation from diverse biomass sources. The extracted cellulose, with a purity range of 83.8–93.9%, was processed into cellulose aerogels (CAs) via a conventional aerogel preparation protocol. The resulting CA exhibited promising adsorption capacities, including 0.2–11.6 mg/g for Na^+^, 4.4–19.9 mg/g for Ca^2+^, 4.15–35.6 mg/g for Mg^2+^, and 1.85–13.3 mg/g for Cd^2+^, as well as 9.7–17.7 g/g for engine oil. These results demonstrate the presence of effective mass transfer channels in the CA, proving that the cellulose’s fibrillation capacity was preserved in the pre-treatment. This study illuminates the potential of this green, straightforward method for preparing aerogels from cellulose derived from waste biomass, with promising applications in wastewater treatment and material recovery.

## 1. Introduction

Water treatment, encompassing both wastewater and seawater management, is of paramount importance in safeguarding human health [1]. Among the diverse techniques of wastewater treatment, adsorption has emerged as a particularly notable method, distinguished by its high efficiency, operational simplicity, and low energy demands [2,3]. Aerogels, with their highly porous structures and abundant activated sites, are regarded as highly promising candidates for water treatment adsorbents [4]. Nevertheless, a significant limitation of aerogels lies in the difficulty of preparation and high cost. In the context of sustainable development of society, renewable raw materials are more environmentally benign and cost-effective solutions. Bio-based materials are derived from renewable biological resources, such as plants, animals, and microorganisms. These materials are gaining significant attention due to their potential to reduce dependency on fossil fuels, lower carbon footprints, and provide sustainable alternatives to conventional materials. Cellulose, a plentiful, cost-efficient, and renewable natural polymer, is a typical linear polysaccharide composed of d-glucan units connected by β-(1, 4) glycosidic bonds [5]. The annual production of cellulose is estimated at 10^11^~10^12^ tons [6,7]. Cellulose is sourced from various waste biomass, such as straw, cotton, flax, wood, and cotton. Particularly, only straw has an annual yield of 700–900 million tons [8]. Due to their affordability and abundant availability, the production of cellulose aerogels (CAs) from waste biomass not only confers economic benefits but also carries environmental implications [9,10].

Currently, CAs derived from waste biomass are being developed and are progressively employed in the adsorption domain [11], mainly for the removal of pollutants such as dyes, pesticides, surfactants, and heavy metal ions, exhibiting impressive adsorption performances [12,13]. Gupta et al. [14] reported that CA derived from date palm leaves exhibited remarkable adsorption capacities of 72.9, 114.4, 92.9, and 123.9 mg/g for arsenic, cadmium, nickel, and zinc ions in water, respectively. Dilamian et al. [15] utilized rice straw to prepare CA, and after hydrophobic modification, it exhibited an impressive oil adsorption capacity of up to 170 g/g. Akhlamadi et al. [16] derived cellulose nanocrystals from waste paper towels, revealing high adsorption capacities ranging from 69 to 168 g/g for various oils and organic solvents. However, traditional methods for extracting cellulose from waste biomass are often complex and involve the use of volatile solvents, oxidants, and strong acids or bases. These substances pose potential risks to the operator, the reactor equipment, and the environment at large.

Against this backdrop, ionic liquids (ILs) have emerged as a potential solution. ILs are considered green solvents due to their low volatility, non-corrosiveness, and ability to dissolve lignocellulosic biomass efficiently. They can break down complex biomass structures, such as lignin and cellulose, through the disruption of hydrogen bonds and other intermolecular interactions, making them a promising alternative to traditional solvents. They can facilitate the degradation of lignocellulose by breaking various connecting bonds such as β-O-4 ether bonds [17], intra- and intermolecular hydrogen bonds [18], π–π bonds, and n–π bonds [19]. Nevertheless, most extensively studied imidazolium-based ILs have certain drawbacks like high viscosity (ranging from 22 to 40,000 cP), toxicity, poor biodegradability, and complex synthesis procedures. The high viscosity could potentially cause operational difficulties, which could be mitigated by diluting with water but decreasing the solubility of biomass when the water content exceeds 2% [20]. Since most of the drawbacks are attributed to their cation structure, our previous work has explored a novel alternative. We discovered that the aqueous solution of a “fully green” amino acid-derived IL [21], cysteine nitrate ([Cys][NO_3_]), can be easily synthesized by simply mixing nitric acid and cysteine at an equal molar concentration. The aqueous solution of this [Cys][NO_3_] has a remarkable ability to remove hemicellulose and lignin from biomass, enabling the extraction of relatively pure cellulose.

In this study, we leverage the innovative use of the amino acid-derived ionic liquid [Cys][NO_3_] to enable efficient cellulose extraction from various waste biomass sources, including wheat straw, corn stalks, rice straw, paper, jute, and cotton. Through a simple hydrothermal treatment in aqueous [Cys][NO_3_] solution, we successfully separated relatively pure cellulose from these biomass materials. The extracted cellulose was utilized to prepare cellulose aerogels, which were systematically evaluated for their adsorption capacity toward metal ions (Na^+^, Ca^2+^, Mg^2+^, Cd^2+^) and engine oil. Additionally, the adsorption of pollutants in actual wastewater was examined to preliminarily validate the feasibility of preparing aerogels using this green method and their practical viability for wastewater treatment or seawater desalination applications.

## 2. Results and Discussion

### 2.1. Effect of Pre-Treatment on Biomass

#### 2.1.1. Microstructure of Biomass

The SEM images of the biomass before and after pre-treatment with [Cys][NO_3_] disclosed the alterations in surface morphology.

**Figure 1:**
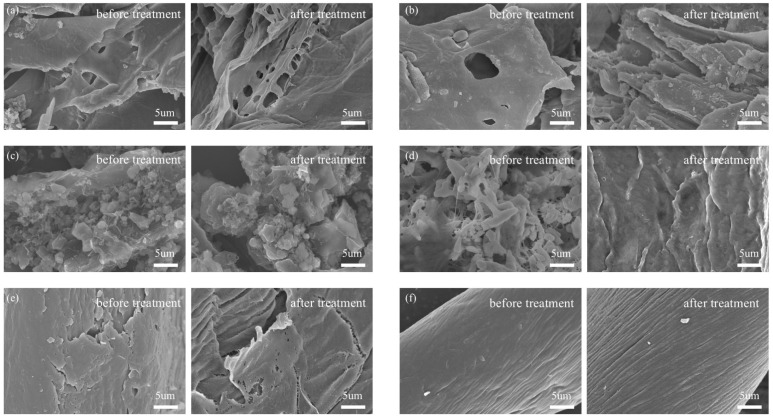
SEM images of (**a**) wheat straw, (**b**) corn stalks, (**c**) rice straw, (**d**) paper, (**e**) jute, and (**f**) cotton. Left: before pre-treatment; right: after pre-treatment.

Before treatment, the wheat straw exhibited a more compact and smoother surface with various fiber layers resisting bacteria during anaerobic digestion. The surface morphology was characterized by a dense arrangement of fibers with minimal porosity, which may have limited the accessibility of the material’s surface area for interactions such as adsorption or digestion. After treatment, the dense structure of wheat straw became loose, disorganized, and incomplete. The smooth surface roughened, particle accumulation decreased, and numerous crevices and voids appeared, exposing the internal tissues (Figure 1a). In the untreated state, corn stalks displayed a complex microstructure with visible pores and grooves, indicative of a natural, rough texture. The pre-treatment process resulted in the stalks exhibiting a broken layered structure and fine particles (Figure 1b). The surface of rice straw before treatment was characterized by a rough texture with visible particles and some porosity. Post-treatment, the surface of rice straw became rough, and fine, crushed, scale-like structures emerged (Figure 1c). The fine flake particles on the surface were more uniformly sized and distributed, possibly due to the removal of the silica layer that originally covered the straw [22]. Conversely, treatment with [Cys][NO_3_] partially disrupted the morphological structure of paper by eliminating the accumulated particulate matter and converting its loose and disordered structure into a compact, orderly, and intact form with a smooth surface (Figure 1d). This is attributable to the minerals and additives in paper. Jute fibers before treatment exhibited a rough texture with visible fibrous structures and pores, contributing to their natural strength. After treatment, the particle accumulations on the surface of jute decreased, but their dense structure became loose and incomplete (Figure 1e), while some cracks and holes emerged due to the dissolution of their components. The surface of cotton had relatively minor changes, with only an increase in surface texture (Figure 1f).

**Figure 2:**
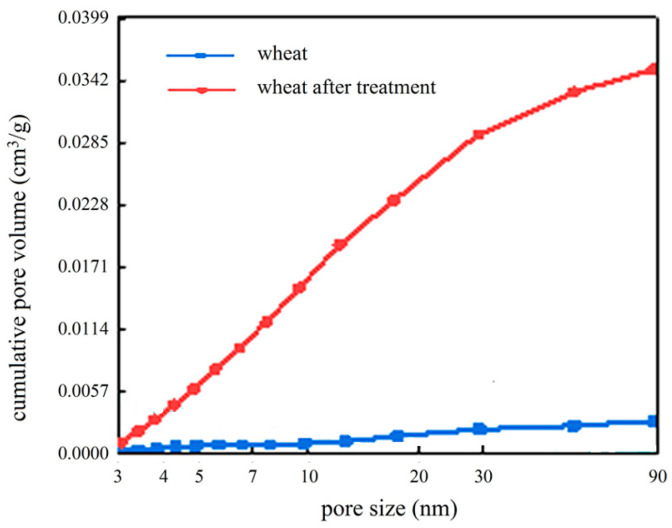
Cumulative pore volume of wheat before and after pre-treatment.

We selected wheat straw as a representative due to its high cellulose content and widespread availability and measured the pore surface area and pore size distribution before and after treatment (Figure 2). After pre-treatment, the BET surface area of wheat straw increased from 1.055 m^2^/g to 14.534 m^2^/g, and the pore volume in the range of 3–90 nm increased from 0.001 to 0.003 cm^3^/g to 0.012–0.033 cm^3^/g.

#### 2.1.2. Chemical Composition of Biomass

Table 1 showing before pre-treatment, the cellulose content of agricultural waste biomass (wheat straw, corn stalks, and rice straw) was relatively lower than that of urban waste biomass (paper, jute, and cotton). After pre-treatment with [Cys][NO_3_], the content of cellulose and organic elements in cotton increased slightly, consistent with the result of SEM. Except cotton, the content of cellulose and organic elements in all of the other biomass increased considerably, indicating that a significant portion of the hemicellulose, lignin, and other organic and inorganic matters were removed by [Cys][NO_3_]. The contents of cellulose ranged from 83.8%~94.0%, suggesting that the obtained materials were relatively pure cellulose.

**Figure 3:**
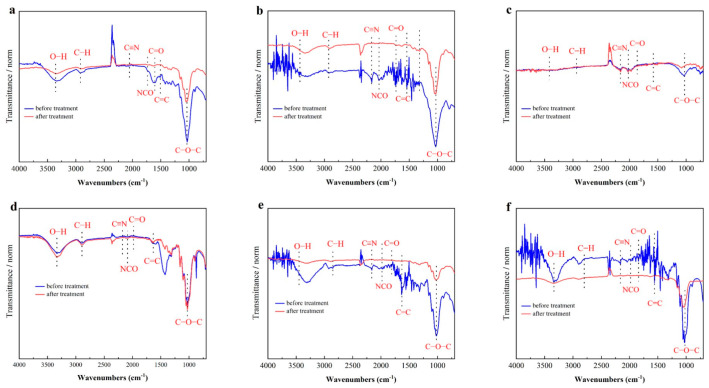
FTIR spectra of (**a**) wheat straw, (**b**) corn stalks, (**c**) rice straw, (**d**) paper, (**e**) jute, and (**f**) cotton. Black lines: before pre-treatment; red lines: after pre-treatment.

Fourier transform infrared spectroscopy (FTIR) analysis was conducted to investigate the chemical bond and functional groups in the six biomasses (wheat straw, corn stalks, rice straw, paper, jute, and cotton) before and after pre-treatment with [Cys][NO_3_]. The FTIR spectra (Figure 3) revealed significant alterations in the functional groups of the biomass materials, providing insights into the mechanism of [Cys][NO_3_] on lignocellulose.

Before treatment, the spectra of agricultural waste biomass (e.g., wheat straw, corn stalks, and rice straw) (Figure 3a, Figure 3b and Figure 3c) exhibited a broad O–H stretching vibration at 3430~3450 cm^−1^, indicative of hydroxyl groups in lignin, hemicellulose, and cellulose, alongside strong hydrogen bonding interactions. After treatment, these O–H peaks underwent a redshift to lower wavenumbers (e.g., wheat straw: 3430 → 3420 cm^−1^; jute: 3420 → 3410 cm^−1^) and displayed reduced intensity, signifying the partial removal of lignin and hemicellulose, which disrupted intermolecular hydrogen bonds and enhanced cellulose purity. In contrast, cotton (Figure 3f)—a cellulose-rich material—showed minimal shifts in O–H peaks (~3445 → 3440 cm^−1^), consistent with its inherently high cellulose content and limited impurity removal [18,23].

The degradation of lignin and hemicellulose was further corroborated by changes in C–H vibrations. Peaks at 2920 cm^−1^ (asymmetric C–H stretching) and 2850 cm^−1^ (symmetric C–H stretching), attributed to methyl and methylene groups in lignocellulosic components, markedly weakened in pre-treated samples. For instance, rice straw exhibited a pronounced decline in these peaks, aligning with the breakdown of lignin’s aromatic side chains. Similarly, the disappearance of the 1740 cm^−1^ band (C=O stretching of ester groups) and the attenuation of the 1630 cm^−1^ peak (C=C aromatic ring vibration) confirmed the cleavage of ester linkages and aromatic structures in lignin, particularly evident in corn stalks and jute [24].

Critical evidence for cellulose depolymerization emerged from the glycosidic bond region. The intensity of peaks at 1160 cm^−1^ (C–O–C stretching in β-1,4 glycosidic bonds) and 898 cm^−1^ (β-glycosidic linkages) decreased significantly in agricultural biomass, such as wheat straw and rice straw, indicating partial disruption of cellulose crystallinity. Concurrently, the near-elimination of the 1060 cm^−1^ peak (pyranose ring in hemicellulose) validated the degradation of hemicellulose. Notably, pre-treated paper (Figure 3d) and jute (Figure 3e) displayed new peaks at 2246 cm^−1^ (–C≡N stretching) and 2171 cm^−1^ (NCO stretching), suggesting covalent interactions between the ionic liquid and cellulose, which may contribute to crosslinking within the aerogel matrix [25,26].

These structural modifications align with the mechanism of [Cys][NO_3_] pre-treatment, which disrupts the lignocellulosic architecture by breaking β-1,4 glycosidic bonds, aromatic rings, and ester linkages. The resultant cellulose, with a purity of 83.8–93.9%, exhibited reduced hydrogen bonding and a more porous morphology, as evidenced by BET surface area increases (e.g., rice straw: 1.055 → 14.534 m^2^/g). The FTIR findings thus provide a molecular-level rationale for the efficacy of [Cys][NO_3_] in sustainable biomass valorization and advanced adsorbent development [27,28].

Overall, the FTIR analysis indicated that [Cys][NO_3_] pre-treatment effectively broke the aromatic rings in lignin, the pyranose ring in hemicellulose, and the β-1,4 glycosidic bonds in cellulose. This treatment reduced the C-O linkages of ester groups and decreased the methyl, methylene, and -OH groups, thereby weakening intermolecular and intramolecular hydrogen bonds and other linkages. The results demonstrated that [Cys][NO_3_] is an effective agent for extracting cellulose from waste biomass, leading to increased cellulose purity.

### 2.2. Properties of Aerogels

**Figure 4:**
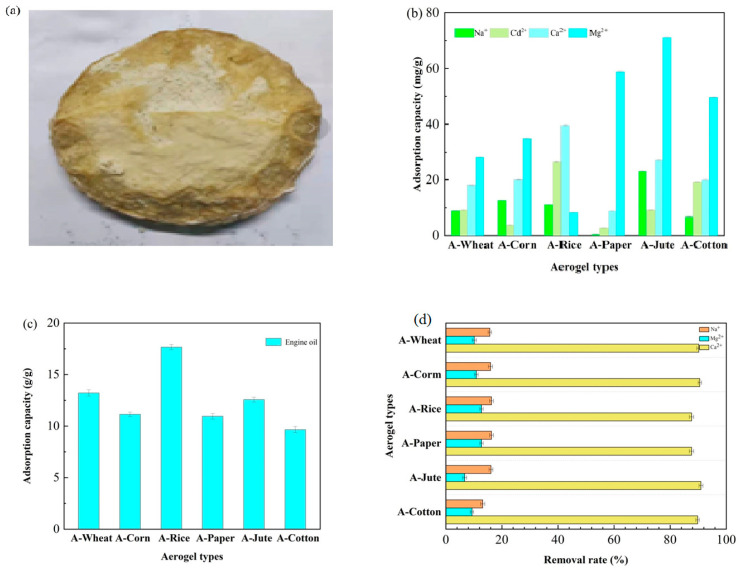
Physical diagram of A-WHEAT aerogel (**a**) and the adsorption capacity of different aerogels for (**b**) metal ions and (**c**) motor oil in real wastewater and (**d**) percentage of pollutants removed.

We still selected A-WHEAT as a representative for pore characteristics. A-WHEAT resembled a fluffy cake (Figure 4a), and exhibited a low density of 0.05 g/cm^3^ and a high porosity of 96.7%, indicating the successful preservation of cellulose’s fibrillation capacity, which is a crucial factor in forming a robust three-dimensional network in aerogel. Disappointingly, after the sample was prepared into A-WHEAT, the surface area dropped to 3.506 m^2^/g, and the pore volume in the range of 3–90 nm became 0.0051–0.0002 cm^3^/g. This might be due to the sample being subjected to high-temperature (120 °C) and high-vacuum conditions for 12 h during the N_2_ adsorption–desorption process as a degassing treatment, which could potentially cause pore structure collapse.

The adsorption capacities (ACs) of different cellulose aerogels for Na^+^, Ca^2+^, Mg^2+^, and Cd^2+^ were 0.2~11.6 mg/g, 4.4~19.9 mg/g, 4.15~35.6 mg/g, and 1.85~13.3 mg/g, respectively (Figure 4b). Notably, A-JUTE had the highest AC for Na^+^ and Mg^2+^ due to its specific surface area and pore structure, while A-RICE had the highest AC for Ca^2+^ and Cd^2+^ due to its higher cellulose purity and chemical structure. In contrast, A-PAPER showed the lowest AC for both Na^+^ and Ca^2+^, whereas A-CORN and A-RICE had the lowest AC for Cd^2+^ and Mg^2+^, respectively. The adsorption capacities were comparable to some adsorbents such as certain biochar [29] but lower than many modified materials.

Since the AC for oil varies greatly depending on the type of oil, we used commercially available engine oil, which is commonly seen in environmental pollution, as the research object. Figure 4c illustrates the ACs of A-RICE, A-CORN, A-WHEAT, A-PAPER, A-COTTON, and A-JUTE, which were 17.7 g/g, 11.2 g/g, 13.2 g/g, 11.1 g/g, 12.6 g/g, and 9.7 g/g, respectively. It is noteworthy that A-RICE exhibited the highest AC, whereas A-JUTE presented the lowest AC. The enhanced adsorption performance of A-RICE for engine oil can possibly be ascribed to multiple factors. These include the wax layer present on the surface of rice straw, the molecular structure of the cellulose polymer, and the physical attributes of the fibers, such as hollow lumens and surface roughness, all of which exert a significant impact on the oil uptake capacity of A-RICE [30,31]. The waxy layer on rice straw surfaces exhibits strong hydrophobicity and lipophilicity, endowing A-RICE with a high affinity for oil, making it particularly suitable for the adsorption and removal of motor oil. Compared to the literature, the ACs of aerogel for oil (including engine oil, petroleum, and vegetable oil) from rice straw, wheat straw, bamboo, filter paper cotton waste, and wood were 13 g/g [32], 6.9~19.2 g/g [33], 20.6 g/g, 19.3 g/g, 13.5 g/g, and 13.7~18.9 g/g [34].

The aerogels were employed for the biochemical tail water of kitchen wastewater, which contained hardly biodegradable organic matters and salts. As depicted in Figure 4d, the removal efficiencies of the six aerogels for Mg^2+^, Na^+^, and Ca^2+^ ranged from 6.7% to 12.7%, 13.2% to 16.3%, and 87.6% to 91.1%, respectively. Remarkably, A-PAPER demonstrated the highest removal efficiencies for both Mg^2+^ and Na^+^, whereas A-JUTE exhibited the highest efficiency for Ca^2+^. The relatively higher removal rate for Ca^2+^ can be ascribed to its lower initial concentration in the wastewater [35,36].

In conclusion, the aerogels prepared from waste cellulose obtained through simple treatment with IL initially demonstrated a certain adsorption capacity (Table 2). The adsorption capacities for Na^+^, Ca^2+^, Mg^2+^, and Cd^2+^ were at a relatively low yet acceptable levels. The adsorption capacity for engine oil was at an ordinary level. This indicates the presence of effective mass transfer channels, due to the formation of macroscopic porous structures. However, the adsorption capacity for refractory organic substances in real wastewater was basically negligible.

## 3. Conclusions

This study successfully demonstrated the use of the amino acid-derived ionic liquid [Cys][NO_3_] for extracting high-purity cellulose (83.8–93.9%) from diverse waste biomass sources, including wheat straw, corn stalks, rice straw, paper, jute, and cotton. The mechanism of biomass deconstruction was elucidated through FTIR analysis, revealing its ability to disrupt the aromatic rings in lignin, the pyranose rings in hemicellulose, and the β-1,4 glycosidic bonds in cellulose, while also reducing inter- and intramolecular hydrogen bonds.

The extracted cellulose was used to prepare cellulose aerogels (CAs), which exhibited promising adsorption capacities for metal ions and engine oil. The adsorption capacities (ACs) of the CA materials were as follows: Na^+^ (0.2–11.6 mg/g), Ca^2+^ (4.4–19.9 mg/g), Mg^2+^ (4.15–35.6 mg/g), Cd^2+^ (1.85–13.3 mg/g), and engine oil (9.7–17.7 g/g). These results highlight the presence of effective mass transfer channels, proving the preservation of cellulose’s fibrillation capacity, which is a crucial factor in forming a robust three-dimensional network in aerogel.

In summary, this study presents a green and scalable method for extracting cellulose from waste biomass and demonstrates the feasibility of using the resulting cellulose to prepare aerogels with moderate adsorption capacities. The findings underscore the potential of this approach for sustainable material development, while future work should focus on optimizing the synthesis of aerogels to enhance their structural and functional properties.

## 4. Materials and Methods

### 4.1. Materials

The test wheat straw, rice straw, and corn stalks were sourced from Lianyungang City, Jiangsu Province. The jute and cotton were obtained from waste fabrics, and the waste paper (recycled paper) was Deli Jiaxuan multifunctional copy paper (No. 3560) after printing.

The biochemical tail water of kitchen wastewater was collected from a sewage treatment plant in Wuxi, China. The concentrations of Na^+^, Mg^2+^, Ca^2+^, and TOC were 6947.3, 514.1, 43.7, and 217.7 mg/L, respectively.

### 4.2. Methods

#### 4.2.1. Pre-Treatment of Materials

L-cysteine and nitric acid were mixed at a molar ratio of 1:1 to become an aqueous solution of ionic liquid with a mass fraction of 75%, i.e., nitric acid–cysteine ([Cys][NO_3_]). The crushed paper, corn stalks, cotton, wheat straw, rice straw, and corn stalks with a diameter of 2 mm were placed on an 80-mesh sieve, first rinsed with tap water, washed sufficiently until the filtrate was colorless, then rinsed with ultrapure water 3~4 times, and finally dried in an oven, weighed, and set aside.

A total of 10 g of dry solid waste and 200 g of amino acid ionic liquid ([Cys][NO_3_]) were added to a conical flask in a mass ratio of 1:20, and the mouth of the flask was sealed with filter paper. The conical flasks were then placed in an autoclave at a set temperature of 110 °C for 180 min.

Remove the conical flask, place it at room temperature, and allow it to cool. Pour the contents into a funnel for filtration. First, wash the treated solid waste materials with deionized water, thoroughly rinsing until the filtrate is colorless. Then, wash with 95% ethanol 2~3 times to obtain the pre-treated solid waste materials. Place them in a 65 °C oven to dry for later use.

#### 4.2.2. Synthesis of Aerogels

A total of 2 g of the pre-treated biomass was added to an aqueous solution of NaOH/PEG-4000 (9:1 wt/wt) and stirred for 5 h to form a homogeneous solution. Then, the cellulose solution was frozen at −15 °C for 12 h and then stirred vigorously for 30 min at room temperature [37].

After freezing again at −15 °C for 5 h, the cellulose solution was regenerated sequentially by (i) placing the frozen sample in 1 v% hydrochloric acid solution for 6 h and repeating this process until an amber-like hydrogel was formed; (ii) soaking the resulting hydrogel in water for 6 h and repeating this process three times to remove the excess chloride ions; and (iii) soaking the hydrogel in tert-butanol for 6 h and repeating this process three times to remove the water from the previous step.

Finally, the synthesized hydrogels were freeze-dried in near vacuum (25 Pa) at −45 °C for 48 h. After depressurization, cellulose aerogels were obtained. The aerogels prepared from six different materials, namely waste paper, hemp, cotton, wheat straw, rice straw, and corn stove, were named A-PAPER, A-JUTE, A-COTTON, A-WHEAT, A-RICE, and A-CORN, respectively.

#### 4.2.3. Static Adsorption Experiments

The mixture of aerogel in the solution of a contaminant was agitated at 25 °C until the concentration of the contaminant no longer changed. The concentration of the organic pollutants was measured using an ultraviolet spectrophotometer (U-2910, Hitachi, Japan), while the concentration of the metal ions was measured using a flame atomic absorption spectrometer (AA7700, Shimadzu, Japan). The adsorption capacity (*Q_e_*) of the aerogel was calculated using the following formula:

AC (mg/g) = (C_0_ − C_e_)/m × V (1)

where C_0_ is the initial concentration (mg/L); C_e_ is the equilibrium concentration (mg/L); V is the solution volume (L); m is the adsorbent mass (g).

#### 4.2.4. Methods for the Determination of Lignocellulose Content

The lignin, cellulose, and hemicellulose contents of the samples were determined using Van’s method for determining cellulose content (hereafter referred to as Van’s method). The samples were washed by a neutral detergent and an acidic detergent to obtain neutral detergent fiber (NDF) and acidic detergent fiber (ADF), respectively. The remaining material after soaking in 75% sulphuric acid with ADF for 3 h included silicate and lignocellulose. It was then burned continuously for 2 h at a set temperature of 600 °C in a muffle furnace, and acidic dystrophic lignin (ADL) was released during the ashing process, and the remaining residual solid was silicate. The contents of hemicellulose, cellulose, and lignin were calculated according to Equations (3), (4) and (5), respectively.

NDF(%) = (Z_1_ − Z_2_)/Z × 100 (2)

where NDF is the neutral detergent fiber (%); Z_1_ is the NDF and ceramic crucible mass (g); Z_2_ is the ceramic crucible mass (g); Z is the specimen mass (g).

ADF(%) = (C_1_ − C_2_)/Z × 100 (3)

where ADF is the acid detergent fiber (%); C_1_ is the NDF and ceramic crucible mass (g); C_2_ is the ceramic crucible mass (g); Z is the sample mass (g).

Hemicellulose(%) = NDF(%) − ADF(%) (4)

Fiber = ADF(%) − Residue from sulfuric acid treatment(%) (5)

ADL(%) = Detritus(%) − Ash(Silicate%) (6)

### 4.3. Characterization

The contents of cellulose, hemicellulose, and lignin in waste paper were determined by an automatic fiber analyzer (A2000i, Ankom, Macedon, NY, USA) using the Van Soest method [38]. The specific pore volume and pore size of the samples were measured based on the N_2_ adsorption–desorption method (TriStarII, Micromeritics, Norcross, GA, USA). The contents of C, H, N, and O elements were determined by an organic element analyzer (Unicube, Elementar, Langenselbold, Germany). The morphology was characterized by scanning electron microscopy (SU8010, Hitachi, Tokyo, Japan). Functional groups of the samples were characterized using FTIR spectroscopy (Nicolet Is50, Thermo Scientific, Waltham, MA, USA) using the KBr disk method in the wavenumber range of 400–4000 cm^−1^. The density of the aerogel (ρ) was calculated using Equation (7), in which m and V represent the mass and volume of the aerogel, respectively [39].

(7) ρ=m/V

The porosity of aerogel was calculated by Equation (8) [40]:

(8) $$porosity=\left(\frac{\rho_{0-}\rho }{\rho_{0}}\right)\times 100\%$$

where $\rho_{0}$ is the skeletal density of cellulose (1.53 g/cm^3^) [41].

## Figures and Tables

**Table 1:** Content of cellulose and organic elements before and after treatment of different biomass.

		Cellulose	C	N	H	O
wheat straw	before	42.8%	32.7%	1.31%	4.39%	31.3%
	after	83.8%	42.8%	1.56%	5.91%	48.9%
corn stalks	before	46.8%	30.9%	1.29%	4.44%	33.5%
	after	85.9%	41.1%	1.55%	5.93%	51.1%
rice straw	before	49.4%	30.4%	1.19%	4.34%	33.1%
	after	93.5%	41.2%	1.54%	5.97%	50.9%
paper	before	62.9%	35.5%	1.31%	6.28%	14.8%
	after	89.5%	40.7%	1.51%	7.40%	24.0%
jute	before	72.8%	33.1%	1.28%	4.42%	31.6%
	after	94.0%	38.2%	1.46%	5.30%	40.5%
cotton	before	89.8%	34.8%	1.30%	4.19%	28.2%
	after	90.8%	34.9%	1.40%	4.27%	28.4%

**Table 2:** Best adsorbing aerogels for different metal ions and engine oils.

Best	Na^+^	Ca^2+^	Mg^2+^	Cd^2+^	Engine Oil
aerogel	A-JUTE	A-PAPER	A-JUTE	A-PAPER	A-RICE
AC	11.6 mg/g	19.9 mg/g	35.6 mg/g	13.3 mg/g	17.7 g/g

## Data Availability

The original contributions presented in this study are included in the article. Further inquiries can be directed to the corresponding author.
